# Galanin Protects Rat Cortical Astrocyte from Oxidative Stress: Involvement of GalR2 and pERK1/2 Signal Pathway

**DOI:** 10.1155/2019/2716028

**Published:** 2019-05-22

**Authors:** Jing Sun, Shu Xu, Hui Li, Liang Li, Zhi-Qing David Xu

**Affiliations:** ^1^Department of Neurobiology, Beijing Key Laboratory of Neural Regeneration and Repair, Beijing Laboratory of Brain Disorders (Ministry of Science and Technology), Beijing Institute of Brain Disorders, Capital Medical University, Beijing 100069, China; ^2^Department of Pathology, Capital Medical University, Beijing 100069, China; ^3^Department of Neurorehabilitation, Beijing Boai Hospital, China Rehabilitation Research Centre, Beijing 100068, China; ^4^Department of Anatomy, Capital Medical University, Beijing 100069, China

## Abstract

The neuropeptide galanin and its receptors have been found to have protective effects on neurons. However, the role of galanin on astrocytes is still unclear. The present study is aimed at investigating the effects of galanin on the viability of cultured rat cortical astrocytes after oxidative stress induced by H_2_O_2_ and possible receptor and signaling mechanisms involved. Treatment of galanin had significant protective effects against H_2_O_2_-induced toxicity in the cultured cortical astrocytes. H_2_O_2_ induced an upregulation of phosphorylated extracellular signal-related kinase1/2 (pERK1/2) in astrocytes, which was suppressed by coapplication of galanin, suggesting an involvement of the pERK1/2 signal pathway in the protective effects of galanin. GalR2 has higher expression levels than GalR1 and GalR3 in the cultured cortical astrocytes, and GalR2 agonist AR-M1896 mimicked galanin effects on the astrocytes, implying that galanin protective effects mainly mediated by GalR2. Meanwhile, galanin had no effect on the A1-type transformation of rat cortical astrocytes. All those results suggest that galanin protects rat cortical astrocytes from oxidative stress by suppressing H_2_O_2_-induced upregulation of pERK1/2, mainly through GalR2.

## 1. Introduction

In the central nervous system (CNS), astrocytes contribute to maintain the homeostasis of the CNS [1]. As a component of blood-brain barrier, astrocytes serve as functional barriers that attract and restrict CNS inflammation [2]. Astrocytes keep the balance between their opposing functions of glutamate uptake and release [3, 4], providing glial cell involvement in the pathophysiology of epilepsy [5]. Astrocytes could drive seizure generation in mitochondrial epilepsy [6], even atypical astrocytes contributed to spontaneous recurrent seizures after diffuse traumatic brain injury [7]. Also, astrocytes contribute to synaptic plasticity, neuronal network oscillations, and cognitive processes, playing a role in Alzheimer's disease [8]. Astrocytes respond to all forms of CNS insults through a process referred to as reactive astrogliosis. This process is a highly heterogeneous state; the function of astrocytes may be harmful or beneficial [9]. Thus, two different types of reactive astrocytes were termed “A1” (harmful) and “A2” (beneficial), respectively, according to neuroinflammation and ischemia [10].

Galanin, a 29-30 amino acid neuropeptide [11], is widely expressed in the CNS [12]. It is involved in many physiological and pathological functions, such as memory, epilepsy, Alzheimer's disease, and depression [13]. So far, three galanin receptors have been cloned, termed as GalR1, GalR2, and GalR3 [13]. Galanin is found coexisting with many classic neurotransmitters, such as 5-hydroxytryptamine in the dorsal raphe nucleus (DR), norepinephrine in the locus coeruleus (LC), and acetylcholine in the medial septal nuclei (MS) [12, 14]. Thus, galanin plays a cotransmission role in the CNS [15]. Many studies have showed that galanin may also have neurotrophic/neuroprotective effects in addition to its neurotransmission role. Its neuron protection functions have been proved from in vitro primary cultured hippocampal neurons to in vivo animal models and transgenic models [16–18]. The neuronal protections of galanin are mediated mostly by GalR2 [17, 18]. However, GalR1 has recently been found to have a protective effect on neurons in the rat hippocampus and ischemic mouse brain [19, 20]. However, little research has been performed to investigate the protective role of galanin on astrocytes. Priller and colleagues found that galanin is able to induce c-fos mRNA in cultured rat astrocytes, providing evidence for the presence of functional galanin receptors on glial cells [21]. In the present study, the effects of galanin on the viability of cultured rat cortical astrocytes after H_2_O_2_-induced oxidative stress as well as the receptor and signaling mechanisms involved were investigated.

## 2. Materials and Methods

### 2.1. Culture of Rat Cortical Astrocytes

Astrocyte cultures were prepared from the cerebral cortex of 1-day-old neonatal Sprague Dawley rats. After decapitation, the brainstem, cerebellum, and diencephalons were removed in cold dissection buffer, the meninges were peeled off, then the brain were minced by scissors, incubated with 0.25% trypsin–EDTA at 37°C for 5 min, filtered through a 200 mesh. Cells were incubated at 37°C in a 5% CO2 for 1 hour in DMEM/F12 supplemented with 10% fetal bovine serum. The culture media were collected, and cells were resuspended in DMEM/F12 supplemented with 10% fetal bovine serum and 1% penicillin/streptomycin and plated on poly-L-lysine-coated 75 cm^2^ flasks at 37°C in a 5% CO_2_ incubator. After about 2 weeks, cultures reached confluence and were shaken at 250 rpm for 18 hr at 37°C to dislodge cells adhering to the astrocyte layer, mainly oligodendrocytes. Secondary astrocyte cultures were established by trypsinizing confluent cultures and subplating onto dishes. In the present study, astrocytes were used at passage 3. When astrocytes were transformed, TNF*α* (30 ng/ml, MCE), IL-1*α* (3 ng/ml, MCE), and C1q (400 ng/ml, BioVision) were added in the medium.

### 2.2. Cytotoxicity Assay

Sensitivities of astrocytes to various chemicals were examined using the Cell-Counting Kit (CCK, Sigma, St. Louis, MO, USA) technique. Astrocytes were plated at a density of 5000 cells per well in 96-well plates. After 24 hr incubation at 37°C in a 5% CO_2_ incubator, culture medium was replaced with new medium and drugs, incubated for an additional 24 hr. 10 *μ*l CCK reagent was added into each well and incubated for 2 hr before reading at a wavelength of 450 nm. The drugs added into each well included several groups, vehicle, H_2_O_2_, H_2_O_2_+galanin, galanin, H_2_O_2_+AR-M1896, and AR-M1896. Absorbances were converted to percentages for comparison with the vehicle group.

### 2.3. Immunocytochemistry Staining

Cells on 25 mm poly-L lysine-coated glass coverslips were rinsed twice with PBS, pH 7.2-7.4, fixed with 4% paraformaldehyde in PBS for 15 min at room temperature, rinsed three times with PBS, incubated with PBS containing 0.3% Triton X-100 for 30 min, blocked in 10% goat serum in PBST for 1 hr, incubated with primary monoclonal anti-GFAP mouse antibody (Sigma, St. Louis, MO, USA) overnight at 4°C, rinsed three times with PBS, incubated with secondary goat anti-mouse Alexa Fluor 488 (Invitrogen, Carlsbad, CA, USA) for 2 hr at RT, rinsed three times with PBS, mounted with glycerin, and examined under confocal microscope (Leica, USA) or inverted fluorescence microscope IX51 (Olympus, Japan).

### 2.4. Western Blot

Astrocytes were washed with PBS, lysed in RIPA lysate containing protease inhibitor cocktail (Applygen, China) and phosphatase inhibitor cocktail (Sigma, USA), and sonicated for 2 minutes. Cell lysates were centrifuged for 20 min at 13000 g at 4°C. Supernatant proteins were separated by SDS-PAGE on 12% gels and transferred onto PVDF membranes. After blocking with 5% nonfat milk in Tris-buffered saline, pH 7.5, containing 0.1% Tween 20 (TBS-T) for 2 hours at room temperature, blots were incubated with primary antibodies in TBS-T overnight at 4°C. The primary antibodies included rabbit anti-pERK1/2 antibody (1 : 1000, Cell Signaling Technology) and mouse anti-Gapdh antibody (1 : 10000, Sigma). Then, blots were washed with TBS-T three times and incubated with horseradish peroxidase-conjugated secondary antibody (goat anti-rabbit or goat anti-mouse) (1 : 5000, China) at RT for 2 hours. Finally, the blots were rinsed and visualized using the enhanced chemiluminescence system (Thermo Fisher Scientific, Rockford, USA) according to the manufacturer's instructions. Optical densities of individual blot were quantified using the ImageJ software. Ratios of pERK1/2 to Gapdh were calculated for each sample, and fold changes were shown compared to the control group.

### 2.5. Reverse Transcription of mRNA

Total mRNA was isolated from culture of rat cortical astrocytes using the Qiagen RNeasy Mini Kits (Qiagen, Hilden, Germany), and mRNA was reverse transcribed using the SuperScript™ III RT reagent (Invitrogen, Carlsbad, CA, USA) according to the manufacturer's manual.

### 2.6. Polymerase Chain Reaction (PCR)

PCR reaction was carried out using the PrimeSTAR® HS DNA Polymerase (Takara, Tokyo, Japan) under the following conditions: 2 min 98°C, 30 cycles of 10 s 98°C and 1 min 68°C, then 10 min 68°C. The primers were listed in Table 1. The identities of the PCR products were confirmed by sequencing.

### 2.7. Real-Time Quantitative PCR (qPCR)

Real-time Quantitative PCR was carried out with SYBR Green (ABI). The total reaction system was 20 *μ*l, 50°C 2 min, 95°C 10 min, 40 cycles for 95°C 15 sec, and 60°C 1 min. Gapdh was set as the internal parameter and the relative mRNA levels were calculated with the 2^−ΔΔCt^ method. The primers were listed in Table 2.

### 2.8. Statistical Analysis

Results were presented as means ± SEM or median (interquartile range). Data were evaluated by one-way ANOVA or nonparametric tests. A value of *p* < 0.05 was considered statistically significant.

## 3. Results

### 3.1. H_2_O_2_-Induced Toxicity in Cultured Rat Cortical Astrocytes

**Figure 1:**
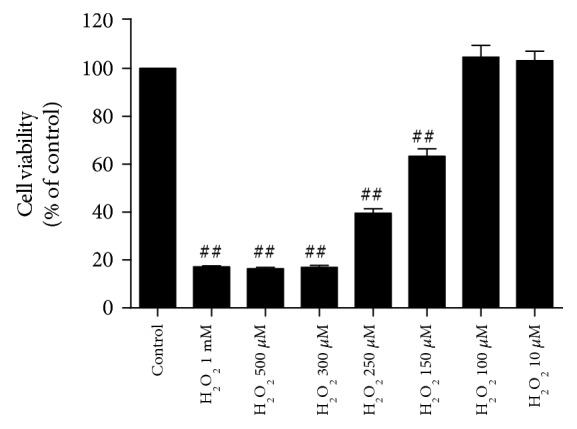
H_2_O_2_-induced toxicity in cultured rat cortical astrocytes. Cell viability was determined by CCK assay. H_2_O_2_ caused a dose-dependent effect on astrocyte viability. Data were presented as mean ± SE. ^#^*p* < 0.05, ^##^*p* < 0.01 vs. control group.

Using CCK assay, we found that the toxic effect of H_2_O_2_ on astrocyte viability was dependent on the concentration of H_2_O_2_ applied (Figure 1). The maximum effect of H_2_O_2_ was the astrocyte viability down to below 20%. Since the astrocyte viability was about 60% when 150 *μ*M of H_2_O_2_ was applied, we chose this concentration to test the effects of galanin in the present study.

### 3.2. The Protective Effects of Galanin against H_2_O_2_-induced Toxicity in Cultured Rat Cortical Astrocytes

**Figure 2:**
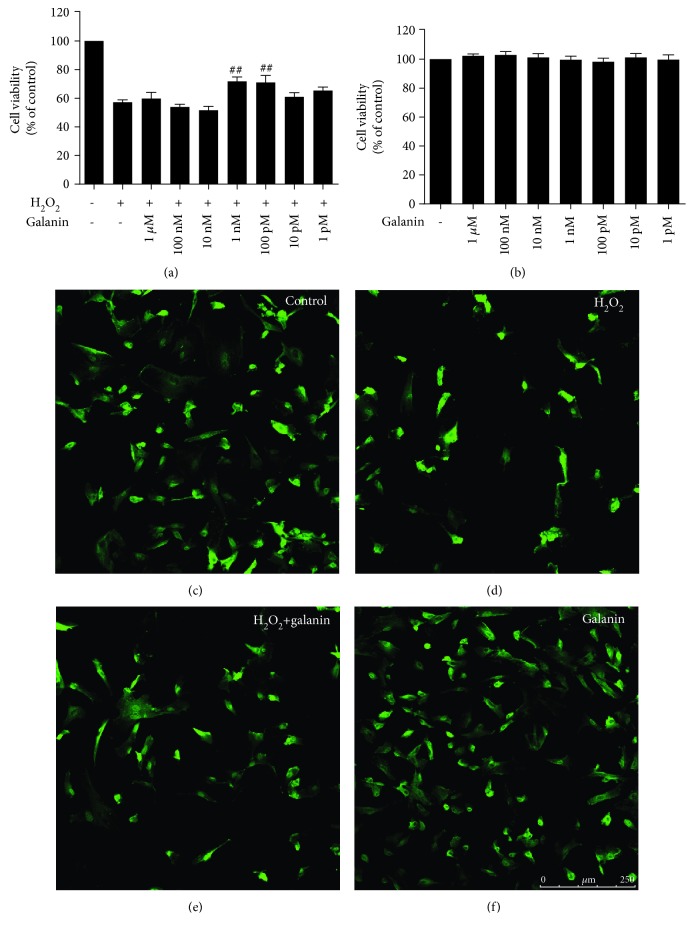
The protective effects of galanin against H_2_O_2_-induced toxicity in cultured rat cortical astrocytes. (a) Using CCK assay, treatment with 1 nM and 100 pM of galanin showed significantly protective effects against H_2_O_2_-induced toxicity; (b) treatment with galanin alone did not have any significant effects on the astrocyte viability compared to the control group; (c–f) galanin reversed the cell death induced by H_2_O_2_. Immunocytochemistry staining of GFAP in different groups: (c) control group, (d) 150 *μ*M H_2_O_2_ group, (e) 150 *μ*M H_2_O_2_+1 nM galanin group, and (f) 1 nM galanin group. Data were presented as mean ± SE. ^#^*p* < 0.05, ^##^*p* < 0.01 vs. H_2_O_2_ group. Bar = 250 *μ*m.

In order to investigate the protective effects of galanin against H_2_O_2_-induced toxicity, cultured rat cortical astrocytes were treated with vehicle, 150 *μ*M H_2_O_2_, 150 *μ*M H_2_O_2_+ galanin with various concentrations (1 *μ*M, 100 nM, 10 nM, 1 nM, 100 pM, 10 pM, and 1 pM), respectively. As shown in Figure 2(a), when the astrocytes were treated with the coadministration of galanin at 1 nM and 100 pM, the cell viabilities were higher as compared to that of the H_2_O_2_ treatment group significantly (*p* < 0.01). Galanin at lower (<10 pM) or higher (>10 nM) concentrations had no significant effects on cell loss induced by H_2_O_2_. Meanwhile, treatment with galanin alone, with various concentrations (1 *μ*M, 100 nM, 10 nM, 1 nM, 100 pM, 10 pM, and 1 pM) did not have any significant effects on the astrocyte viability (Figure 2(b)). After immunocytochemistry staining of GFAP, we found that the numbers of astrocyte were much less when treated with H_2_O_2_ compared to the control group (Figures 2(c) and 2(d)), while galanin (1 nM) rescued partly the loss of astrocytes (Figure 2(e)). Galanin alone did not change the number of astrocytes significantly (Figure 2(f)).

### 3.3. Involvement of pERK1/2 in the Protective Effect of Galanin against H_2_O_2_-Induced Toxicity

**Figure 3:**
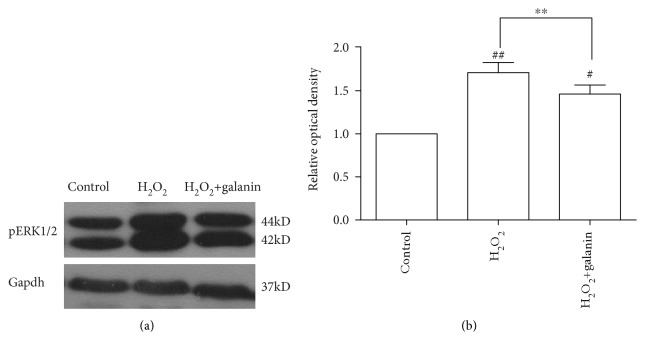
Galanin suppressed the H_2_O_2_-induced upregulation of the pERK1/2 protein level. (a) Representative blots of pERK1/2 in control, 150 *μ*M H_2_O_2_, and 150 *μ*M H_2_O_2_+1 nM galanin groups; (b) ratios of pERK1/2 to Gapdh were calculated and compared. Data were presented as mean ± SE. ^#^*p* < 0.05, ^##^*p* < 0.01 compared with the control group; ^∗^*p* < 0.05, ^∗∗^*p* < 0.01 compared with the H_2_O_2_ group.

To determine the involvement of pERK1/2 in the protective effect of galanin against H_2_O_2_-induced toxicity, we performed the western blot experiment. The results showed that the pERK1/2 protein levels were significantly increased in the 150 *μ*M H_2_O_2_ group compared to the control group (*p* < 0.01). Coapplication of 1 nM galanin significantly suppressed the H_2_O_2_-induced upregulation of the pERK1/2 level (*p* < 0.01). However, the pERK1/2 protein level was still higher in the H_2_O_2_+galanin group compared to the control group (*p* < 0.05) (Figure 3).

### 3.4. Involvement of GalR2 in the Protective Effect of Galanin against H_2_O_2_-Induced Toxicity

**Figure 4:**
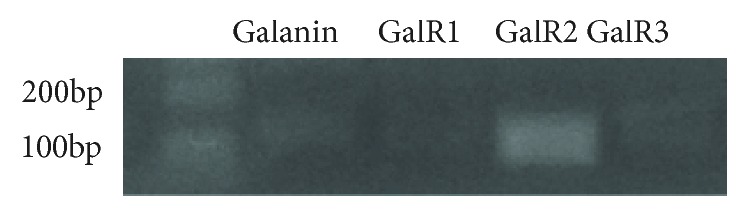
Moderate expression of GalR2 and weak expression of GalR1 and GalR3 in cultured rat cortical astrocytes. The expression of galanin, GalR1, GalR2, and GalR3 was detected by RT-PCR.

The mRNA expression of galanin, GalR1, GalR2, and GalR3 was detected in the cultured rat cortical astrocytes, respectively. As shown in Figure 4, expression levels of GalR2 were moderate while levels of GalR1 and GalR3 were very weak, suggesting mainly GalR2 existed in rat cortical astrocytes.

**Figure 5:**
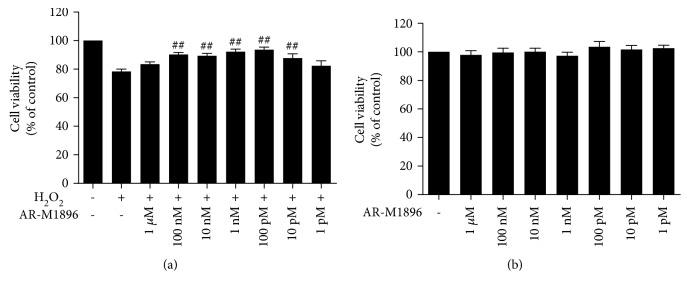
The protective effects of AR-M1896 against H_2_O_2_-induced toxicity in cultured cortical astrocytes of rats. (a) Using the CCK technique, treatment with 100 nM-10 pM of AR-M1896 showed significant protective effects against H_2_O_2_-induced toxicity; (b) treatment with AR-M1896 alone did not have any significant effect on the astrocyte viability compared with the control group. Data were presented as mean ± SE. ^#^*p* < 0.05, ^##^*p* < 0.01 vs. the H_2_O_2_ group.

To investigate which subtype(s) of galanin receptor mediated the galanin-induced protective effects against H_2_O_2_ toxicity in cultured rat cortical astrocytes, GalR2 agonist AR-M1896 [22] was used. The cultured astrocytes were treated with vehicle, 150 *μ*M H_2_O_2_ and H_2_O_2_+AR-M1896 with various concentrations (1 *μ*M, 100 nM, 10 nM, 1 nM, 100 pM, 10 pM, and 1 pM), respectively. As shown in Figure 5(a), coadministration with AR-M1896 at concentrations from 100 nM to 10 pM (*p* < 0.01) showed significantly higher astrocyte viability compared with H_2_O_2_ alone. But treatment with AR-M1896 alone at various concentrations tested above did not change astrocyte viability measured with CCK assay (Figure 5(b)). These results suggest that mainly GalR2 mediates the protective effects of galanin in cultured rat cortical astrocytes.

### 3.5. No Modulation Effects of Galanin on Levels of A1 transcripts in Cultured Cortical Astrocytes

**Figure 6:**
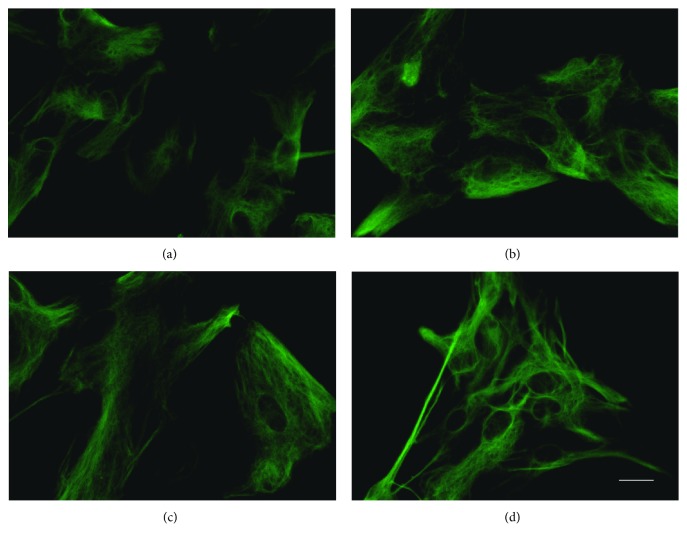
Cultured cortical astrocytes activated by combined application of TNF*α*, IL-1*α*, and C1q. The cell body turned hypertrophy. Immunocytochemistry staining of GFAP was detected in astrocytes of different groups: (a) control group, (b) TNF*α*+IL-1*α*+C1q group, (c) TNF*α*+IL-1*α*+C1q+galanin group, and (d) galanin group. Bar = 20 *μ*m.

**Figure 7:**
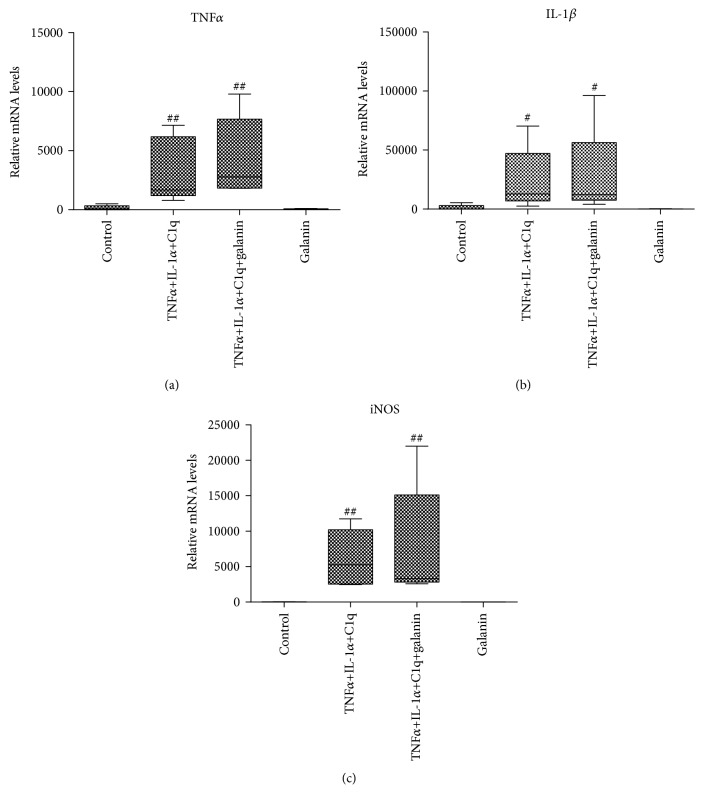
TNF*α*, IL-1*β*, and iNOS mRNA of cultured cortical astrocytes elevated by combined application of TNF*α*, IL-1*α*, and C1q. Application of galanin did not change A1 phenotype. The relative transcriptional levels of TNF*α*, IL-1*β*, and iNOS genes were detected with qPCR. Data were presented as median (interquartile range). ^#^*p* < 0.05, ^##^*p* < 0.01 vs. the control group.

It has been reported that astrocytes could be transformed to A1-type reactive astrocytes, which contribute to neuron death [10]. In order to investigate if galanin is able to revert A1 reactive astrocytes back to resting astrocytes, IL-1*α*, TNF*α*, and C1q were applied to induce A1 reactive astrocytes [10]. As shown in Figure 6, after the treatment, the body of astrocytes turned hypertrophy. Meanwhile, A1 transcripts such as TNF*α*, IL-1*β*, and iNOS in cultured rat cortical astrocytes were robustly elevated (Figure 7 and Table 3). However, coapplication of galanin had no significant effects on the upregulation of A1 transcripts (Figure 7, Table 3).

## 4. Discussion

Galanin has been considered as a neurotransmitter/neuromodulator in the nervous system [23]. Meanwhile, accumulated evidences indicate that galanin also plays a neurotrophic/neuroprotective effect to subsets of neurons in the peripheral and central nervous systems [16]. For example, in both in vivo and in vitro models of injury, more hippocampal neuronal cell death was observed in the galanin knockout mice and less hippocampal neuronal cell death was observed in the galanin-overexpressing transgenic mice, compared with the WT controls [24]. Galanin also inhibits the neurotoxicity induced by amyloid-beta in primary cultured hippocampal neurons from human, rat, and transgenic animal [25, 26].

In the present study, for the first time, we demonstrated that galanin, at concentrations ranging from 100 pM to 1 nM, had significant protective effects on H_2_O_2_-induced cell death of cultured rat cortical astrocytes. Exogenous H_2_O_2_ treatment induces reactive oxygen species (ROS) generated intracellularly. When ROS accumulation exceeds cellular capacity, the resulting oxidative stress leads to astrocyte death [27]. H_2_O_2_ had been shown to enhance the phosphorylation of protein kinase ERK in astrocytes [28]. Although the activation of ERK1/2-MAPK has been considered as a signaling for cell survival, growth, and proliferation, it has also been shown that inhibiting the pERK signal pathway reduced astrocyte death induced by oxidative stress [29]. In the present study, the upregulation of pERK1/2 induced by H_2_O_2_ was partly reversed by galanin, suggesting that galanin suppressed the H_2_O_2_-induced toxicity in astrocytes of rats through suppressing the activation of pERK1/2. It should be noted that galanin has also been reported to activate ERK signal in neurons [30]. Thus, the signaling mechanisms of galanin might be different in different cells.

GalR1 and GalR2 are widely expressed in the central nervous system while the expression of GalR3 is limited [31, 32]. It has been demonstrated that the neuroprotective effects of galanin are mediated through its receptors. Thus, galanin has its neuron protection functions through GalR2 [17, 18, 24] or GalR1 [19, 20]. Our study here showed that the GalR2 mRNA expression was moderate while the GalR1 and GalR3 mRNA expression were very low in the cultured rat cortical astrocytes. Moreover, GalR2 agonist AR-M1896 mimicked the protective effects of galanin in the astrocytes. All those results suggest that GalR2 is involved in the protection of cortical astrocytes. This is consistent with previous studies in neurons that GalR2 mediates the protective effects of galanin [18, 33]. Interestingly, galanin had its effect only at concentrations ranging from 100 pM to 1 nM, and AR-M1896 had its effect only at concentrations ranging from 10 pM to 100 nM. The bell-shaped dose responses of galanin has been reported as a critical concentration window in earlier studies [26]. Moreover, we also seen AR-M1896 had broader effective concentration than galanin in the current study. The mechanisms underlying the bell-shaped dose response are still unclear. One possible explanation is galanin might act on different subtype receptors with different binding affinity. However, it needed to be further investigated.

A recent study shows that astrocytes are transformed to A1 reactive type when treated with a combination of IL-1*α*, TNF*α*, and C1q in vitro [10]. A1-type reactive astrocytes are considered to be neurotoxic and contribute to neuron death after acute CNS injury. For example, A1-type astrocytes have been proved taken a role in the neuroinflammation of traumatic spinal cord injury [34]. Block of A1 astrocyte conversion by microglia is neuroprotective in models of Alzheimer's disease or Parkinson's disease [35, 36]. Therefore, modulating the harmful effects of A1-type astrocytes could be an important stratagem for the CNS disease treatment. However, galanin had no effect on A1-type transformation of the cultured rat cortical astrocytes in the present study.

## 5. Conclusion

Galanin protected rat cortical astrocytes from H_2_O_2_-induced cell death by suppressing the upregulation of pERK1/2, mainly mediated by GalR2.

## Figures and Tables

**Table 1:** PCR primers.

Primer name	Primer sequence
Rat galanin	Forward: CACATGCCATTGACAACCAC
	Reverse: AACTCCATTATAGTGCGGACG

Rat GalR1	Forward: TCGGGACAGCAACCAAAC
	Reverse: TGCAGATGATTGAGAACCTTGG

Rat GalR2	Forward: GCCGCCATCGGGCTCATCTG
	Reverse: GTCGAGGTGCGCTCCATGCT

Rat GalR3	Forward: ACAGATCTCTTCATCCTCAACTT
	Reverse: GTGAGGTAGATGAGCAGATGTAC

**Table 2:** qPCR primers.

Primer name	Primer sequence
Rat iNOS	Forward: TGGAGCGAGTTGTGGATTG
	Reverse: GTGATGTCCAGGAAGTAGGTG

Rat TNF*α*	Forward: CTTCTGTCTACTGAACTTCGGG
	Reverse: CTACGGGCTTGTCACTCG

Rat IL-1*β*	Forward: GCAGGCTTCGAGATGAAC
	Reverse: GGGATTTTGTCGTTGCTTGTC

Rat Gapdh	Forward: GACCACCCAGCCCAGCAAGG
	Reverse: TCCCCAGGCCCCTCCTGTTG

**Table 3:** The relative transcriptional levels of TNF*α*, IL-1*β*, and iNOS genes in astrocytes.

	Control	TNF*α*+IL-1*α*+ C1q	TNF*α*+IL-1*α*+C1q+galanin	Galanin
TNF*α*	85.14 (321.11)	1672.21 (4983.80)	2781.99 (5841.54)	81.13
IL-1*β*	165.75 (2852.47)	12848.98 (40156.28)	12153.73 (48747.71)	156.90
iNOS	2.56 (28.90)	5250.60 (7667.45)	3292.36 (12294.74)	1.78

## Data Availability

The data used to support the findings of this study are available from the corresponding author upon request.
